# LED based real-time survival bioassays for nematode research

**DOI:** 10.1038/s41598-018-30016-5

**Published:** 2018-08-01

**Authors:** Satish Kumar Rajasekharan, Chaitany Jayaprakash Raorane, Jintae Lee

**Affiliations:** School of Chemical Engineering, Yeungnam University, Gyeongsan, 38541 Republic of Korea

## Abstract

Nematode bioassays are extensively conducted worldwide, either for screening anthelmintic drugs or for assessing the toxicity of drug candidates. Recently, the US Environmental Protection Agency mandated the use of invertebrate models including nematodes especially *Caenorhabditis elegans*, for toxicity testing as an alternative to rodent models. The significance of nematode bioassays in the biological sciences is escalating, but no standardized protocol is available to assess nematode mortality in a liquid medium. Manual counting under white light is the only approach currently practiced, which exhibit large variabilities and false positive results. Here, we describe an innovative counting strategy that employs light-emitting diode (LED) technology. We found that the nematodes stopped moving under white light (360–760 nm) when administered with sub-lethal dosage (LC_50_) of a toxic drug, whereas they responded rapidly to blue (450–490 nm) and ultraviolet (UV) (100–400 nm) LED lights. Furthermore, paralyzed nematodes responded in less than 5 seconds to a LED pulse. The response to the LED stimulus was distinctively noted in *C*. *elegans* dauers, which squirmed away from illuminated sites within seconds. LED produced an incoherent beam, and uniformly distributed light across the sampling area. In conclusion, this method is more accurate than the conventional counting techniques, and enables us to differentiate paralyzed and dead nematodes virtually in real-time. Furthermore, this technique would appear to be suitable for incorporating a motion-sensor based automated system.

## Introduction

The phylum Nematoda contains some of the most ancient invertebrates on earth^1^. Their replication is rapid, and importantly they do not have grave predators^2^. Nematodes can disseminate within an ecosystem and infect diverse living organisms, which serve as hosts or carriers^3^. Most importantly, dauers tend to develop rapid resistance to drugs, climatic and chemical stressors^4^.

When exposed to toxic chemicals or nematicides, nematodes are paralyzed, and some even die, but certain stages like dauers are more resilient to toxicity stress^5^. Nematodes exposed to a sub-lethal dosage of toxic chemicals often become inactive but are not dead^6^, for example, ivermectin paralyzes and kills microfilariae in human blood circulation, but does not eradicate adult worms^7^. Practically, it is difficult to differentiate between live and dead nematodes in the laboratory. However, researchers use the lack of motility or inactivity as an indication of nematode mortality, but sometimes nematodes cease motion and appear to be dead during molting or when exposed to sub-lethal dosages, and this causes errors in manual assessment of nematode mortality^6^.

*Caenorhabditis elegans* is viewed as a universal model in studies on molecular biology, genetics, or in the toxicities of drugs and chemicals^8,9^. *C*. *elegans* models provide an alternative to the use of rodent models^8^. Also in Frank R. Lautenberg, Chemical Safety for the 21st Century Act, 2016 mandates that the U.S. Environmental Protection Agency explore possibilities of using “non-vertebrate” models for toxicity testing^8^.

Nematode bioassays are extensively used in several biological sectors, including agricultural, forestry, veterinary, molecular biology, neurotoxicology, genotoxicology, and molecular genetics^8^. Several publications have reported experiments on *C*. *elegans* and documented toxicity levels based on worm survival which is a rough approximation without detailed methodologies. Recently, bacteria or yeast infected *C*. *elegans* have been used to investigate the *in vivo* anti-hyphal activities of chemicals^10^. Nematode toxicity analyses are often conducted in an M9 liquid buffer^11,12^ while several *in vitro* anthelmintic screenings are conducted in sterile distilled water^13^. Nematode mortality is assessed by microscopic observation and manual counting under white light (360–760 nm), or by touching worms with a platinum wire, which do not yield reliable data in a liquid media.

There is no standardized protocol devised to count the nematodes in a liquid medium^14^. Only a few laboratories implemented automated techniques based on flow cytometry (COPASTM Biosort, fluorescence-activated cell sorting), due to cost and inadequate facilities^15–17^. Lack of standardized protocols significantly impedes the determination of LC_50_, IC_50_, and EC_50_ values of test drugs and causes a wide range of intra- and inter-laboratory variability and repeatability. Here, we devised a facile real-time microscopic method using LED stimuli for counting and differentiating between live and dead nematodes.

## Results and Discussion

**Figure 1:**
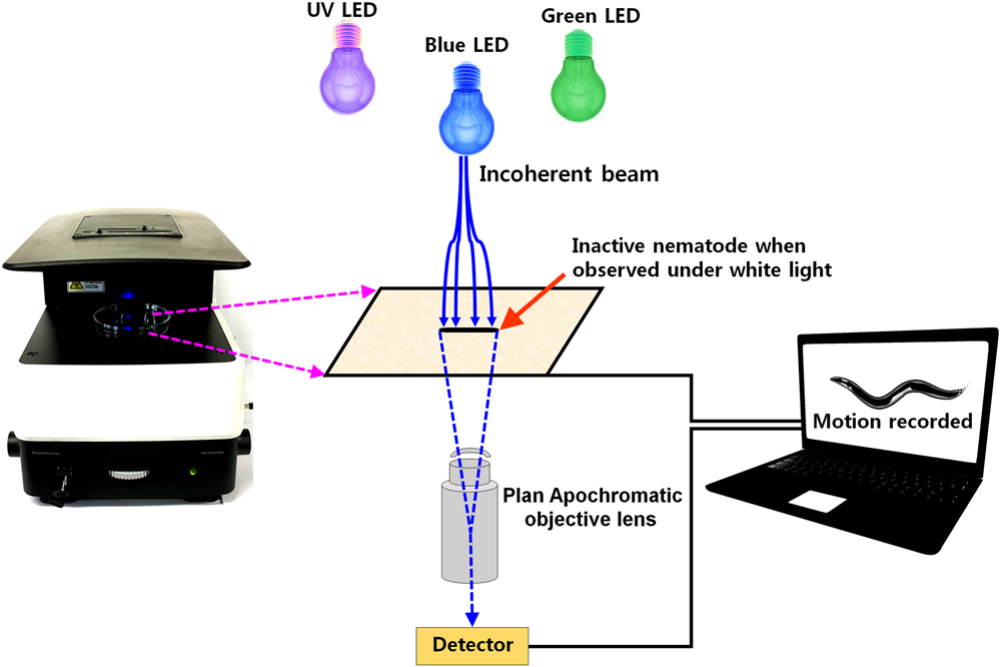
Optical setup for real-time microscopic monitoring of paralyzed nematodes. The system consists of an iRiS™ Digital Cell Imaging System (Logos Biosystems, South Korea) equipped with three color LED cube filters. The sample is illuminated by LED light, which is captured by the objective, and subsequently recorded in real-time using a monitor attached to the imaging interface.

Nematode bioassays are frequently performed in a liquid medium by manually counting the live or dead nematodes using visible light. Here, we described an improved method of nematode counting that employs LEDs. The technique relies on the instant responses of nematodes to the dissipation of heat induced by the LEDs. The microscopic imaging interface used is presented in Fig. 1.

**Figure 2:**
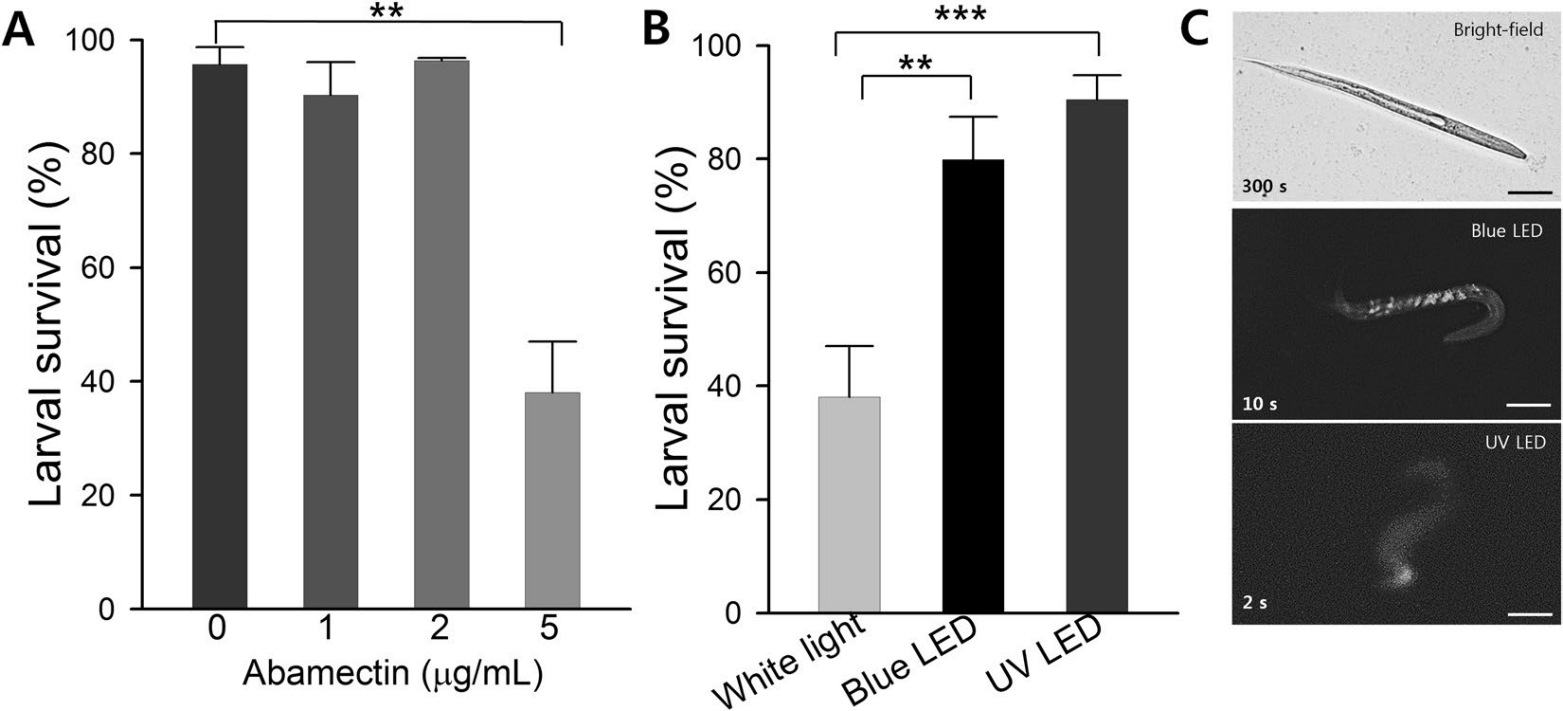
Effect of white light and LED lights on *C*. *elegans* juveniles. (**A**) Estimation of the LC_50_ value of abamectin against *C*. *elegans* juveniles (Graphs are plotted as the means ± SEMs. **P < 0.01 vs. the non-treated controls), (**B**) estimation of the survival rates of juvenile exposed to different light sources (Graphs are plotted as the means ± SEMs. **P < 0.01 vs. the white light controls, and ***P < 0.001 vs. the white light controls), and (**C**) a *C*. *elegans* juvenile visualized under three different light sources (nematodes responded to exposure in 300 s for white light, 10 s for blue LED, and in 2 s for UV LED light). Scale bar: 10 µm.

**Figure 3:**
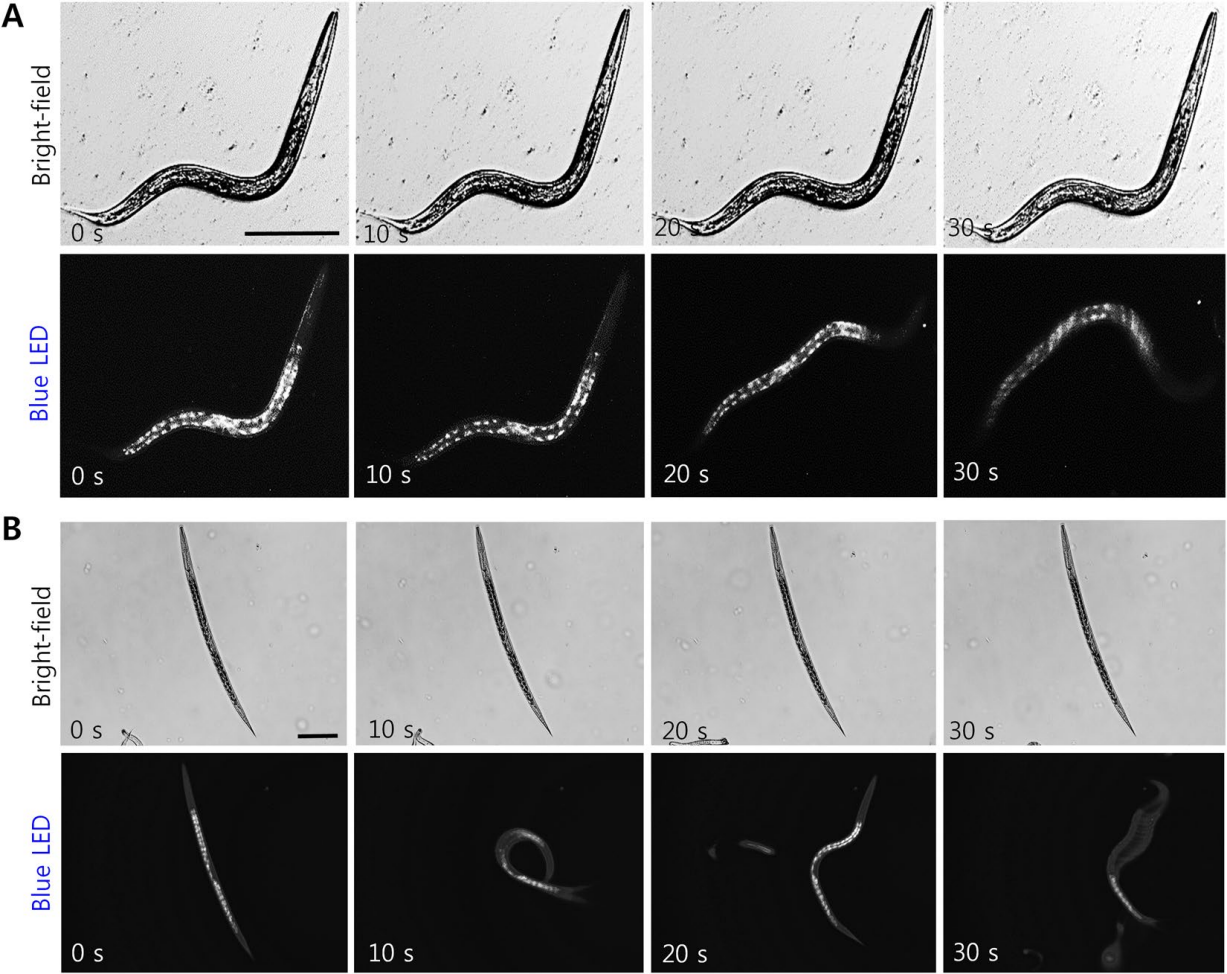
Time-lapse imaging of alive but inactive *C*. *elegans* exposed to a sub-lethal dosage of abamectin. (**A**) An inactive adult *C*. *elegans* visualized under white or blue LED illumination. The nematode exhibited squirming patterns when exposed to blue LED, (**B**) shows the dauer stage under white light capturing its unresponsiveness, but under blue LED light it responded rapidly, and moved from the illumination spot. Scale bar: 50 µm.

LED bioassays on *C*. *elegans* produced consistent results. Initially, synchronized *C*. *elegans* juvenile stages were treated with abamectin. Using regular microscopic counting methods with white light, the sub-lethal dose of abamectin was estimated as 5 µg/mL (Fig. 2A). Few nematodes were inactive, and to appeared dead under white light when treated with abamectin at sub-lethal dosage. Mean survival percentage was 38 ± 9% under normal light. But, when treated nematodes treated with blue LED and UV LED light, mean survival percentages were 80 ± 8% (an error rate of ~40%) and 90 ± 4% (an error rate of ~50%), respectively (Fig. 2B). Nematodes rapidly responded to blue and UV LED lights in 10 and 2 seconds, respectively (Fig. 2C). At a sub-lethal dosage, nematodes behaved differently under white light by remaining immotile for a considerable period, so they were recorded as dead. However, these nematodes responded rapidly to LED stimuli with motion detected within 2 seconds under UV LED exposure. Time-lapse images were acquired to confirm the inactivity of adult nematodes. The adult *C*. *elegans* treated with abamectin (5 µg/mL) shown in Fig. 3A, indicates the nematode did not respond to white light (360–750 nm). Subsequently, we assessed and recorded the motions of this inactive *C*. *elegans* when exposed to a short ten-second pulse of blue LED light. It is also important to note that nematodes survive harsh treatments, and are rendered inactive which makes them appear dead under the experimental conditions. The dauer shown in Fig. 3B remained motionless after exposure to white light for several minutes. But, when exposed to blue LED light, rapid thrashing movements were observed after 10, 20, and 30 seconds of illumination. The responses of dauers to a green LED (510 nm) and UV LED light were also assessed. The dauers did not respond to green LED, but showed rapid flexing when exposed to UV LED light (Supplementary Fig. S1). Their response time probably depends on the dosage of the drug administered, although generally inactive nematodes showed movements within 2 seconds after exposure to UV LED light. During time-lapse imaging, the LED light source was automatically turned off every 10 seconds while capturing the image. Nematodes that underwent ecdysis, a slow process of shedding its old cuticle, were also examined under LED lights. We observed that LED lights triggered rapid movements in these nematodes as well (Supplementary Fig. S2).

**Figure 4:**
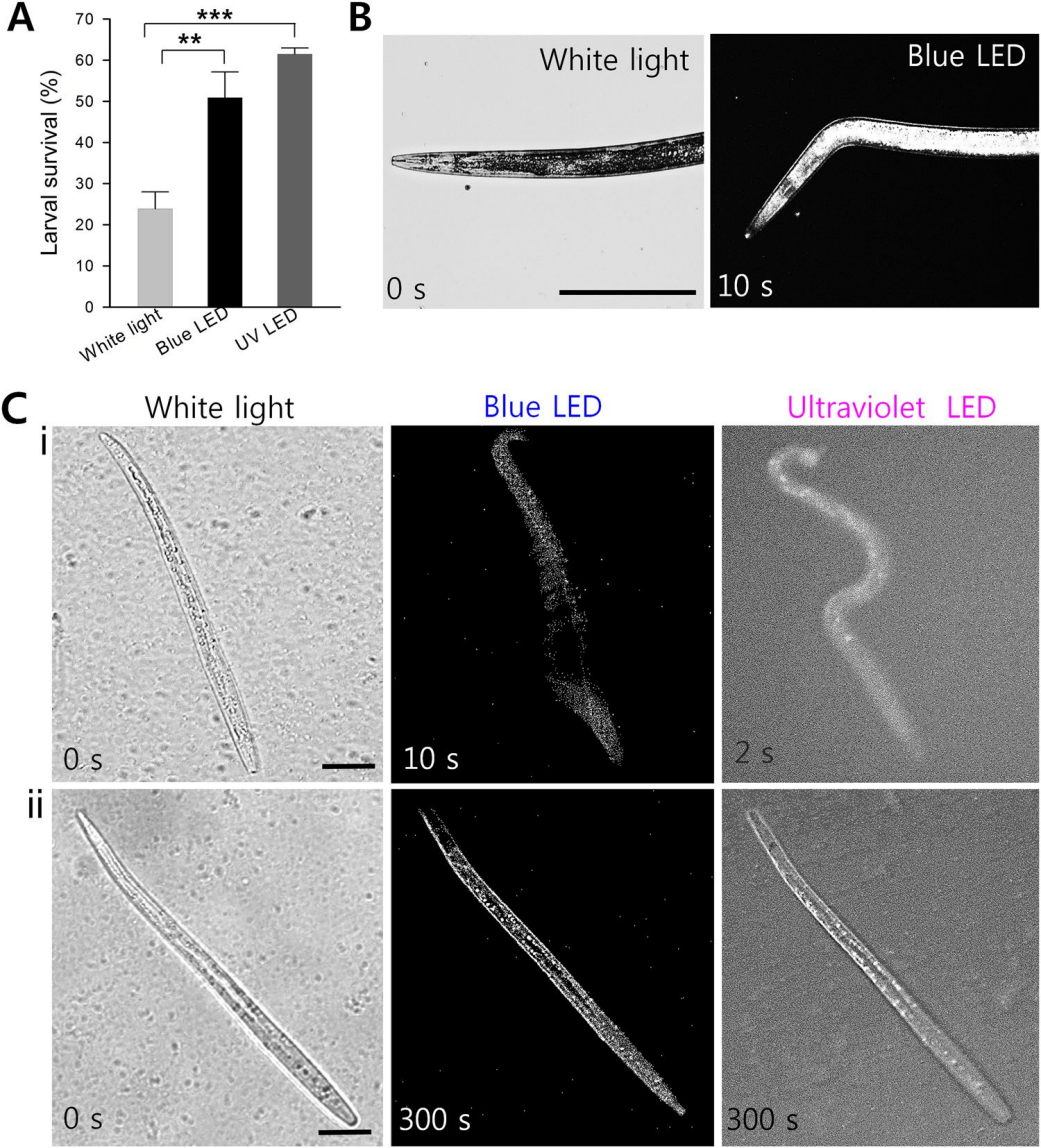
Effect of white light, blue and UV LED light on *B*. *xylophilus* adults and juveniles treated with a sub-lethal dosage of abamectin. (**A**) Survival percentages of J2 stage nematodes treated with abamectin (10 µg/mL) as determined by counted under white light and blue LED light (Graphs are plotted as the means ± SEMs. **P < 0.01 vs. the white light controls, and ***P < 0.001 vs. the white light controls), (**B**) adult *B*. *xylophilus* responding to LED illumination, (**C**) differentiation of (i) live and (ii) dead J2 *B*. *xylophilus* based on exposure to blue or UV LED light (Under white light, both dead and paralyzed J2 worms appear dead). Scale bar: 50 µm.

To examine the reliability of the LED based nematode bioassays, we tested the effect of abamectin (10 µg/mL) on *B*. *xylophilus* (another parasitic nematode), using white light or LED lights, to determine the differences between worm variances. The count for synchronized J2s under regular white light indicated a mean survival rate of 24 ± 4%, whereas under blue LED light the mean survival rate was found to be 51 ± 2%, which translate to an error rate of about 27% (Fig. 4A). The experimental groups treated with abamectin were exposed to UV LED indicated survival rates of these J2s were 61 ± 2%, that is, an error rate of ~37%. The results indicate that manual counting with white light is inaccurate and leads to false positive recordings^6,8^. We demonstrated the difficulty of distinguishing between paralyzed and dead nematodes with white light (Figs 4C i & ii, and 5B). Hence, the paralyzed nematodes might be counted as dead under the conventional counting process. On the other hand, our LED-based counting strategy was found to reduce the inadequacies of the conventional white light method. Figure 4B shows an adult *B*. *xylophilus* responding to LED lights. In Fig. 4C, we show a J2 stage *B*. *xylophilus* treated with a sub-lethal dosage of abamectin. Abamectin is known to kill the instar stages of nematodes and paralyze the adults, so J2 is the best stage for analysis. The paralyzed J2 stage was exposed to blue LED for 300 seconds to record any movement. Under white light, J2s (paralyzed and dead) did not move and appeared dead, while under LED illumination, motions were recorded and blurred images were obtained due to nematode movements, while clear images were obtained for dead nematodes (Fig. 4C). The response times of paralyzed nematodes under blue LED and UV LED lights were 10 and 2 seconds, respectively. All other stages of *B*. *xylophilus* responded similarly to LED stimuli exposure.

**Figure 5:**
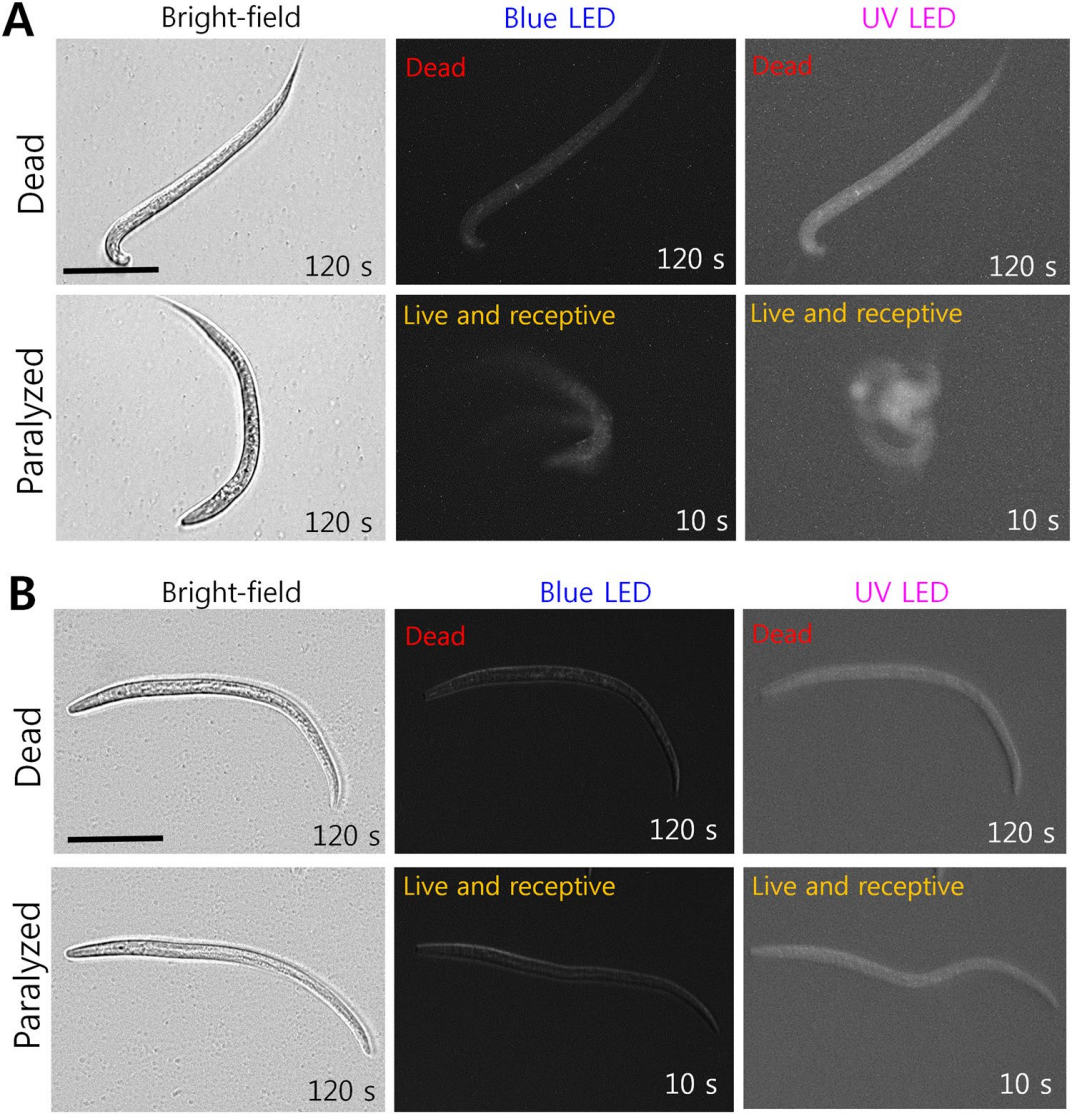
Effect of white, blue and UV LED lights on *C*. *elegans* and *B*. *xylophilus* larval stages treated with sodium azide imaged after recovery process. (**A**) *C*. *elegans* larval and (**B**) *B*. *xylophilus* larval stages were treated with 10 mM (650 µg/mL) sodium azide for a 24 h period before commencing with the recovery process. The figures for both sets indicate that dead nematodes did not respond to exposure to any of the light stimuli, while paralyzed nematodes in both case showed response to blue LED light, and more rapid response to UV LED light, but did not respond to white light exposure. Scale bar: 50 µm.

Assays with other nematicides such as sodium azide, ivermectin, and levamisole were conducted on both *C*. *elegans* and *B*. *xylophilus* larval stages. Sodium azide treatment resulted in instant paralysis of both the nematodes. Sodium azide is an anesthetic agent, and a respiratory inhibitor causing both the nematodes (*C*. *elegans* and *B*. *xylophilus*) not to respond to LED stimuli. After a 24 h, the nematodes in the sodium azide solution were removed and suspended in sterile distilled water or an M9 buffer solution for 30 minutes, and counted under UV LED stimulus (Supplementary Fig. S3). The nematodes treated with 50 mM (3250 µg/mL) sodium azide did not recover and were considered dead, which is in line with the results of a previous study^18^. The nematodes that survived (still paralyzed or partially paralyzed) in 10 mM (650 µg/mL) sodium azide treated groups began to respond to LED stimuli, while the dead ones were still unresponsive (Figs 5 and S4).

**Figure 6:**
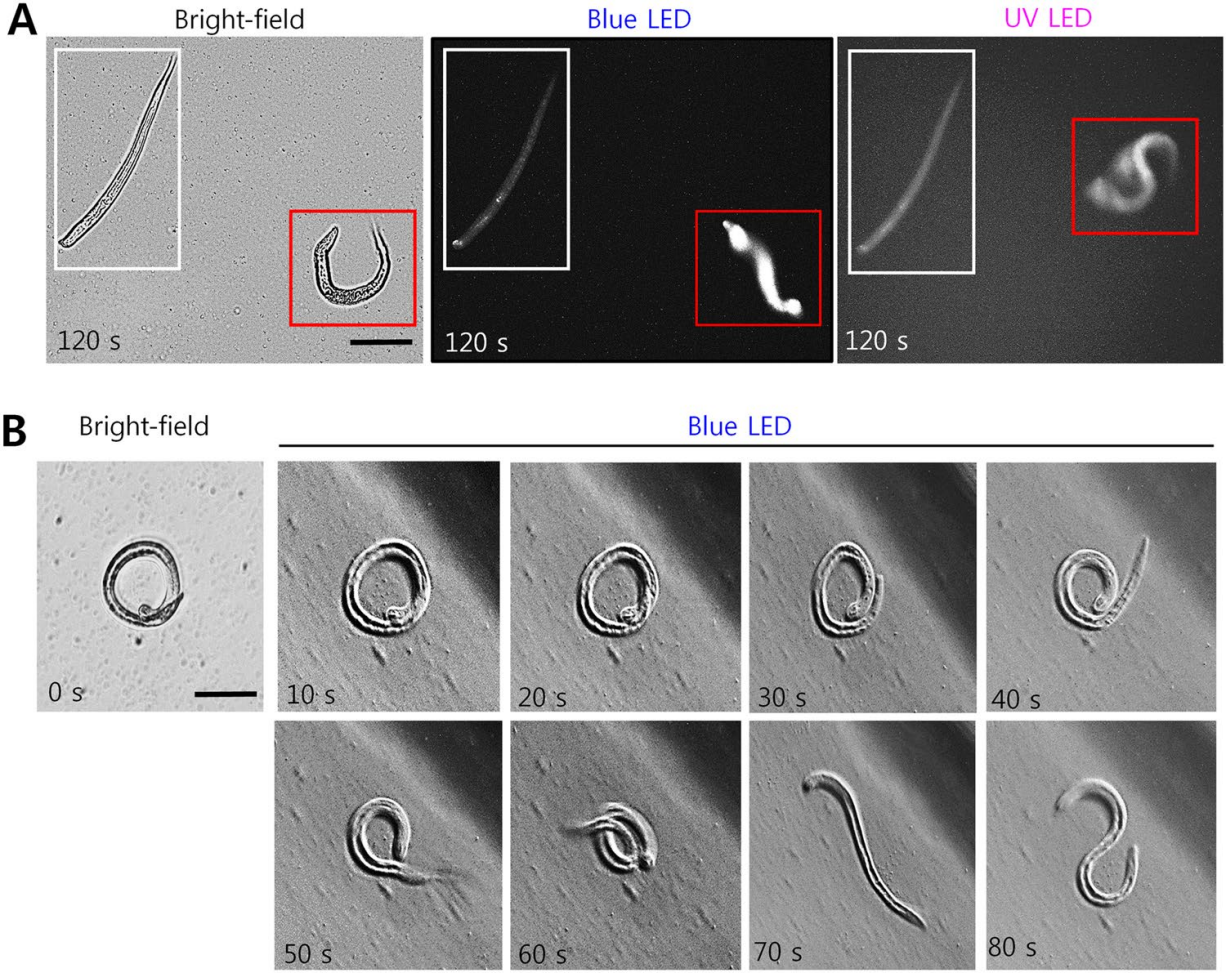
Effect of white light, blue and UV LED light on *C*. *elegans* and *B*. *xylophilus* larval stages treated with levamisole. (**A**) A representative single-frame image showing a dead (white box) and paralyzed (red box) *C*. *elegans* larval stage and its response to white, blue and UV LED lights, and (**B**) a representative image showing a sequence of time-lapse images showing the motion of a paralyzed *B*. *xylophilus* larval stage in response to a blue LED light. Scale bar: 50 µm.

Ivermectin (0, 10, 25, and 50 µg/mL) treated nematodes revealed similar phenotypes like abamectin treatments. Both the drugs belonged to macrocyclic lactone anthelmintics used for treating parasitic nematodes^19^. When tested at lower concentrations (25 µg/mL), nematodes resembled straight dead conformations (Supplementary Fig. S5). Further, levamisole treatment paralyzed the nematodes but did not kill them at the tested concentrations. Most of the nematodes were coiled or showed curly phenotypes (Supplementary Fig. S4). Like sodium azide, levamisole (1–10 mM) is often used to immobilize *C*. *elegans* for microscopic imaging^20^. We show that paralyzed nematodes (*C*. *elegans* and *B*. *xylophilus*) treated with levamisole (1 mM (204 µg/mL)) also recovered well after 24 h and responded to LED stimuli (Fig. 6A and B). Importantly, a few dead ones were intact and motionless, thus differentiating the paralyzed from the dead (Fig. 6B).

Levamisole and sodium azide are drugs that paralyze the nematodes at a sub-lethal dosage and do not necessarily kill them altogether, suggesting that a few of the paralyzed nematodes can recover, when suspended in M9 buffer. It is established that sodium azide, levamisole, and tricaine are anesthetic drugs that stop motility in the worms, and can be subsequently rescued form the anesthetic drugs by transferring them to a fresh M9 media for one hour or less^21,22^. Our LED based platform can reliably distinguish dead nematodes from paralyzed nematodes, thus minimizing the source of potential errors in counting.

Chemical testing on parasitic nematodes is a leading area of research in the field of parasitology and nematology for developments of eco-friendly nematicides. *C*. *elegans* serves as a prominent invertebrate model for toxicity assessments and reduces requirements for rodents. In fact, there is a report indicating that *C*. *elegans* shares significant genomic similarities with rodents^23^. Recently, *C*. *elegans* has been used as an alternative to rodents as a biomedical tool for disease modeling, drug discovery, and toxicity assessments^11,24^. Recently, a research group developed a straightforward counting protocol to minimize the potential sources that may introduce errors^8^. This group standardized the nematode counting methods in a liquid media by minimizing the potential variability sources, which included the priming of pipette tips, shaking of media, sampling location, and operator-to-operator variances. Here we revealed the influence of different drug dosages on nematodes behavior towards LED lights which is also a potential source of variability faced during nematode counting methods.

The behavioral patterns of nematode stages are difficult to understand. Flat and straight nematodes are considered dead during conventional counting. Nematodes in early stages of molt also remain stiff and appear dead. As proof-of-concept, we show that flat and straight nematodes either cease motion during molting (Supplementary Fig. S2) or remain inactive when exposed to sub-lethal dosages of toxic chemicals^6^, but exposure to LED light (Fig. 2B) trigger nematodes to revive or at least move. Nematode staining provides a reliable alternative for differentiating live and dead nematodes during counting, but sample staining in liquid culture is laborious, and increases the degree of experimental difficulties and errors^8^. Staining often brings the live or inactive nematodes under stress. On the other hand, LED based counting provides rapid, accurate, and reliable results. Also, we show the effects of few standard nematicides with different mode of actions namely abamectin and ivermectin (glutamate-gated chloride ion channel (GluCL) agonists^25^), levamisole (nicotinic acetylcholine receptors (nAChRs) agonist^26^), and sodium azide (respiratory inhibitor^18,22^) on two different nematode models (*C*. *elegans* and *B*. *xylophilus*) to validate our findings.

Illumination with blue or UV LEDs induced automatic responses in nematodes. Healthy nematodes moved swiftly when exposed to LED light stimuli to avoid exposure. Previously it was demonstrated that laser ablation effectively kills nematode cells on skin^27^. When a laser microbeam is fired on a specimen, it is thought that the extreme heat and pressure generated denature proteins and DNA^27^. Similarly, prolonged LED exposure has also been shown to cause harmful effects on *C*. *elegans* and to cause retinal damage in rats^28–30^. It has also been reported that *C*. *elegans* possess LITE-1 photoreceptors which are sensitive to laser and UV light stimuli which are responsible for inducing negative photo-tactic responses^31^.

**Figure 7:**
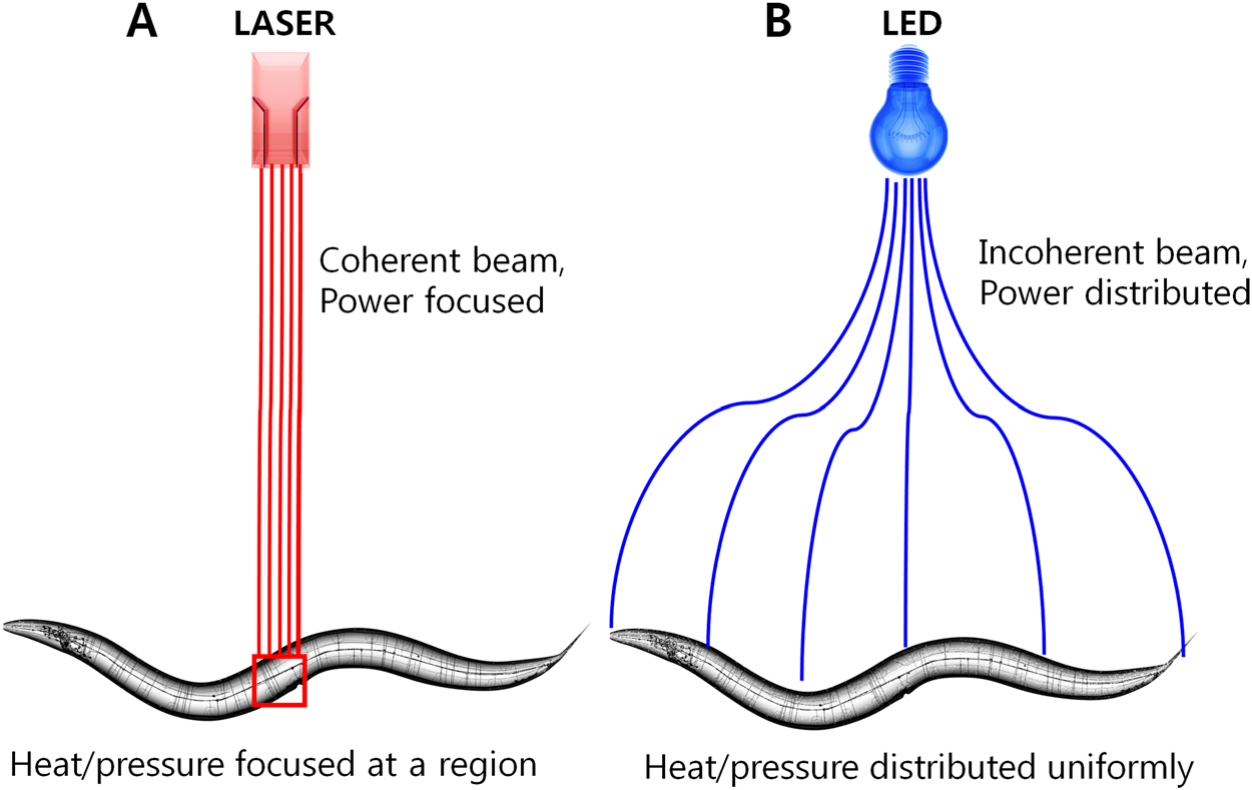
Mechanism of LED and LASER illumination. (**A**) A microbeam of coherent laser light hits a specified target within a nematode, and (**B**) an incoherent beam of LED light illuminates the entire nematode as such.

LED light differs from laser light concerning its coherence (Fig. 7). LED light illuminates specimen by distributing light uniformly across the sample area. The power distribution of LEDs cover a large area and affect several local tissues on the nematodes. The power distribution of LED light is advantageous for live/dead nematode counting, as samples containing ~50 nematodes or more per field can be counted accurately in a short timeframe. The power produced by LED light is sufficient to make the inactive nematodes move from the illumination point. Nematodes response to LED and other noxious stimuli is still a grey area and have to be explored further.

## Future Perspective and Conclusion

**Figure 8:**
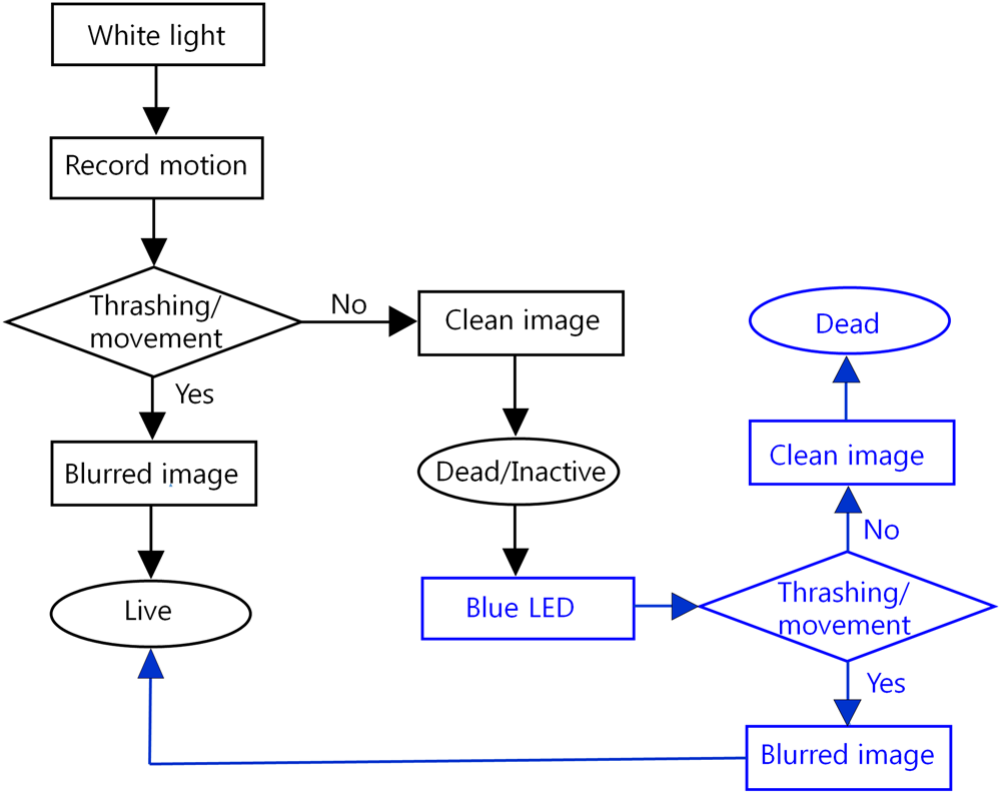
A flow chart describing the protocol of an automated nematode counting system. Black arrows denote white light pulse and the blue arrows denote blue LED pulse.

LED based nematode counting can be performed automatically using algorithms and softwares to identify moving nematodes. The work-flow of this type of motion technology presented in Fig. 8 involves the use of image acquisition software equipped with a real-time motion sensor. This system can be also implemented without white light illuminations as well. Microscopic methods have been devised to detect the motion of small molecules^32^, and several automated methods have been devised to detect nematode thrashing^33^. In fact, high-throughput automated devices like ‘Wormwatcher’ utilize optical microscopic techniques^34,35^. The present study demonstrates the need to merge such techniques with LED illumination.

## Materials and Methods

### Ethics statement

All the experiments were approved by the Ethical Committee of Yeungnam University, Gyeongsan, Republic of Korea and the methods were carried out as per the guidelines of the Ethical Committee of Yeungnam University.

### Tested nematicides

The commercial nematicides, abamectin (Purity-98.7%), levamisole (Purity- 98%), and ivermectin (Purity-98%) were purchased from Sigma (USA). Sodium azide (Purity-97%) was purchased from Duksan Pure Chemicals Co. Ltd (Republic of Korea). Abamectin and ivermectin were dissolved in dimethyl sulfoxide (DMSO), while levamisole and sodium azide were dissolved in sterile distilled water. 0.1% DMSO or sterile distilled water served as the negative control. For nematode bioassay a sub-lethal dosage (LD_50_) of abamectin was used (5 µg/mL for *C*. *elegans* and 10 µg/mL for *Bursaphelenchus xylophilus*).

### *C*. *elegans* culture conditions

*C*. *elegans* wild-type Bristol strain N2 was provided by Professor Eleftherios Mylonakis from Brown University. The strain was maintained on nematode growth medium (NGM) with *E*. *coli* OP50 as feed at 25 °C, and synchronization was also performed as previously described^36^. *C*. *elegans* in NGM plates were collected and bleached with 2% sodium hypochlorite and 0.5 N sodium hydroxide to obtain eggs, which were allowed to hatch to the L1 stage in M9 buffer and then transferred to fresh NGM plates containing *E*. *coli* OP50 lawns. Dauers (morphologically characterized by a slender appearance and pointed tail) were obtained by passaging the L1 stage in a minimal feed medium. Alternatively, *C*. *elegans* eggs were collected in M9 buffer, washed, and transferred to 96-well microtitre plate. Once the eggs settled to the bottom of the plates, the wells were washed repeatedly with M9 buffer to remove any other worms and replaced with fresh M9 buffer, and incubated at 25 °C for 24 h for hatching process. Synchronized *C*. *elegans* larval stages were thus obtained, using abamectin (0, 1, 2, 5, and 10 µg/mL), ivermectin (0, 1, 2, 5, 10, and 25 µg/mL), sodium azide (0, 10 mM (650 µg/mL), and 50 mM (3250 µg/mL))^18^ and levamisole (0, 10, 25, 50, 100, and 200 µg/mL), and incubated at 25 °C for a 24 h period to estimate the LD_50_ and LD_90_ values.

### *Bursaphelenchus xylophilus* and *Botrytis cinerea* culturing

Pinewood nematodes (*Bursaphelenchus xylophilus* (supplied by Professor Hanhong Bae, Yeungnam University)) were sub-cultured by inoculation onto potato dextrose agar (PDA) plates containing fully grown mycelium of the neurotropic fungus *Botrytis cinerea*, as previously described^13^. The nematodes were allowed to reproduce and proliferate on plates for 7–8 days. The plates were then washed with sterile distilled water and the nematodes were further diluted to obtain approximately 100 nematodes per 100 µL. Initially, toxicity testing was performed using mixed stages of nematodes.

### *B*. *xylophilus* J2 mortality assay

J2s mortality assays were assessed with slight modification to the method previously described^13^. Briefly, adult nematodes were collected and transferred to microtitre plates, and a day later, nematodes were selectively removed using a pipette and discarded. Eggs, which adhered to plate bases, were resuspended in sterile distilled water and allowed to hatch. The J2s thus obtained were used for mortality assays. Synchronized J2s (~50 nematodes) were treated with abamectin at a sub-lethal concentration (LD_50_) and incubated at 22 °C for 24 h. Additionally, synchronized the larval stages were treated with ivermectin (0, 10, and 25 µg/mL), sodium azide (0, 10, and 50 mM) and levamisole (0, 10, 25, 50, 100, and 200 µg/mL) to estimate the LD_50_ and LD_90_ values. The experiments were conducted three times and images were acquired using an iRiS™ Digital Cell Imaging System. The survival rate of the J2s was determined by counting the live nematodes, and dead nematodes under white light (350–760 nm), blue LED (450–490 nm), and ultraviolet (UV) LED (100–400 nm) light stimuli. The error percentages were calculated by comparing the nematode survival rates ascertained under white light, blue LED light, and UV LED light, respectively.

### Microscope and imaging interfaces

A simple fluorescent microscope equipped with a camera, a monitor, and integrated image analysis software (The iRiS™ Digital Cell Imaging System, Korea) was used for real-time monitoring. The microscope offered three fluorescence illumination functions such as blue, green and UV, provided by high power, long-lasting LEDs and hard-coated optical fibers. The LED filter cubes in the microscope provide maximum illuminations with uniform distributions. Nematodes exposed to a nematicides (abamectin, ivermectin, sodium azide, and levamisole) were placed in the microscopic stage and initially illuminated with white light to capture bright-field images. Subsequently, images were acquired using time-lapse images (one image per 10 s) captured over on or 2 min, and then following specimens were exposed to LED light (blue (450–490 nm), green (495–570 nm) and UV (100–400 nm)) to visualize nematode movement. The acquired images were either viewed at 40X (4X objective and 10X eyepiece), 100X (10X objective and 10X eyepiece), and/or 200X (20X objective and 10X eyepiece) magnification.

### Data availability

All data generated or analyzed during this study are included in this published article (and its Supplementary Information files).

## Electronic supplementary material

Supplementary information
