# Supplementary material for: LED based real-time survival bioassays for nematode research
Source: Sci Rep. 2018 Aug 1;8:11531. doi: 10.1038/s41598-018-30016-5 (PMC6070477; doi:10.1038/s41598-018-30016-5)

Supplementary Information

LED based real-time survival bioassays for nematode research

Satish Kumar Rajasekharan, Chaitany Jayaprakash Raorane, and Jintae Lee*

School of Chemical Engineering, Yeungnam University, Gyeongsan, 38541, Republic of Korea

***Corresponding author**:

E-mail: jtlee@ynu.ac.kr Phone: 82-53-810-2533. Fax: 82-53-810-4631.

Running Title: LED Based Real-Time Nematode Bioassays

Supplementary Figure S1. Effect of various LED lights on *C. elegans* dauer stage. The dauer was captured after 300 s under bright-field and green LED to which it did not respond, while rapid response was noted when captured under UV LED (2 s) and blue LED (10 s). Scale bar: 100 µm.

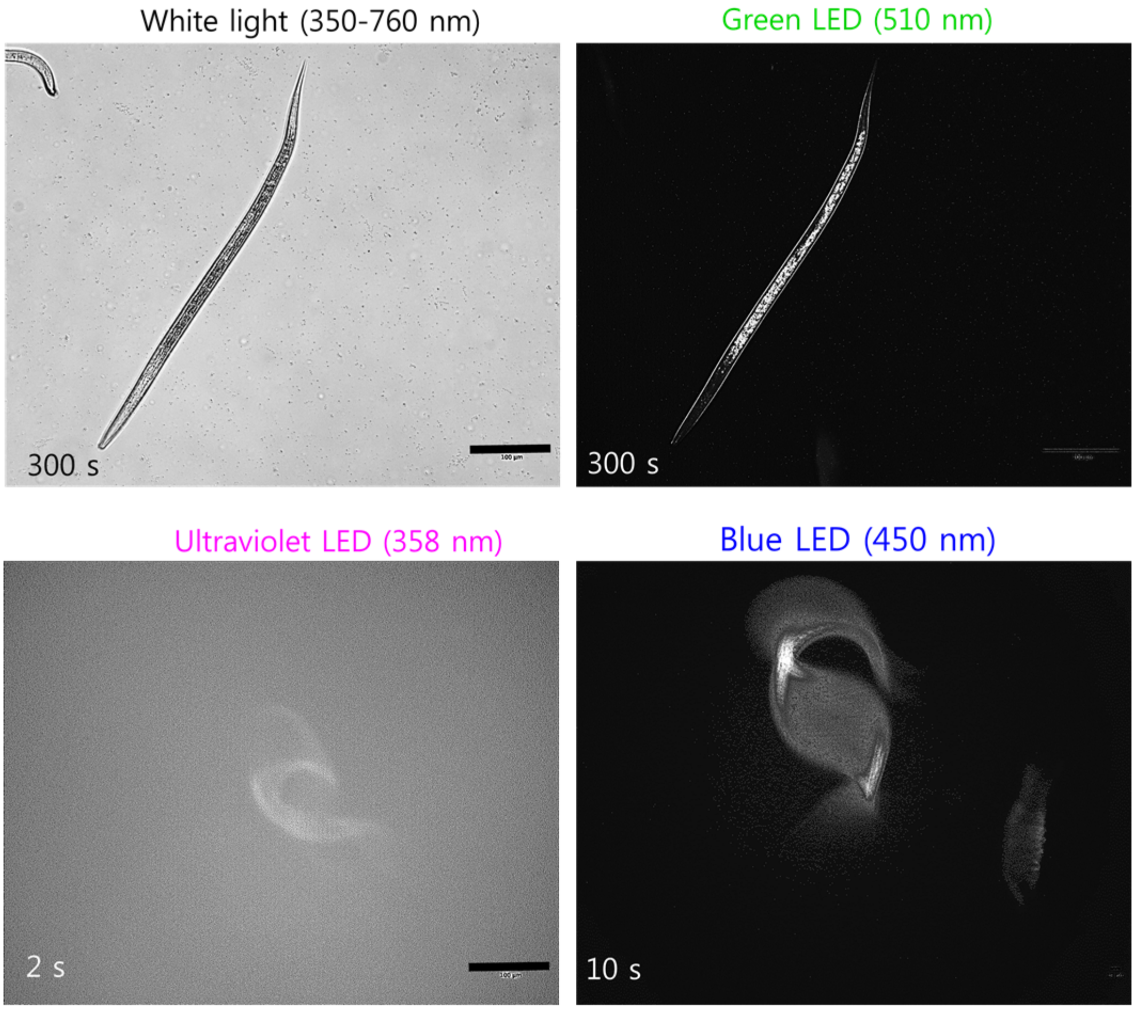

Supplementary Figure S2. Effect of blue LED light on a molting J2 stage *C. elegans*. During molting, the nematode sheds its external skin, and process is gradual with no active motion recorded. Under LED stimuli, rapid response was noted with in a few seconds. Scale bar: 50 µm.

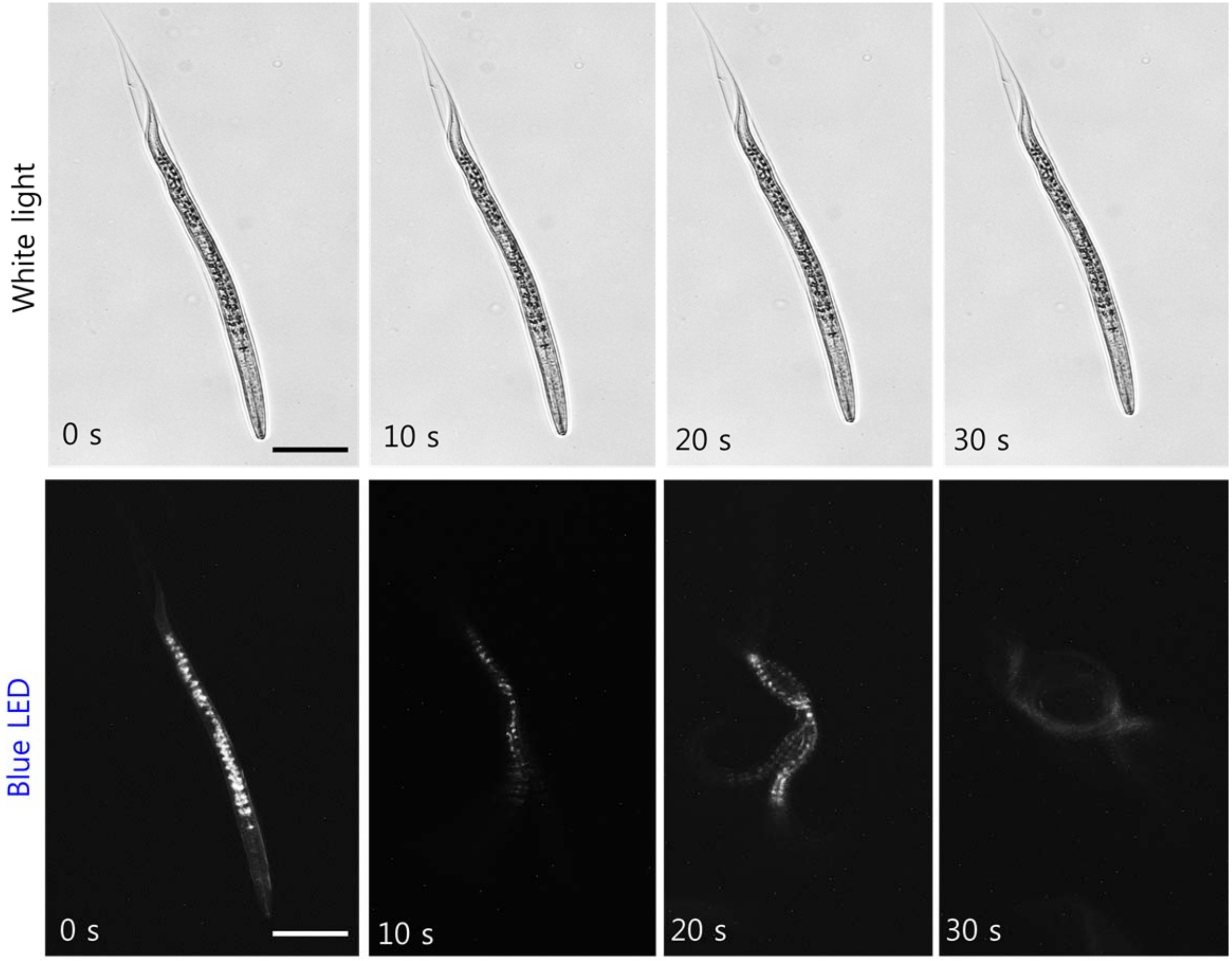

Supplementary Figure S3. Effect of sodium azide on *C. elegans* (A) and *B. xylophilus* (B) larval stages. After 24 h the nematodes were removed from sodium azide solution a nd maintained in sterile distilled water or M9 buffer for 30 min for recovery. Then the nematodes were counted under UV LED stimulus. Graphs are plotted as the means ± SE Ms. ** *P*<0.01 vs. the non-treated controls, N/D: Not detected.

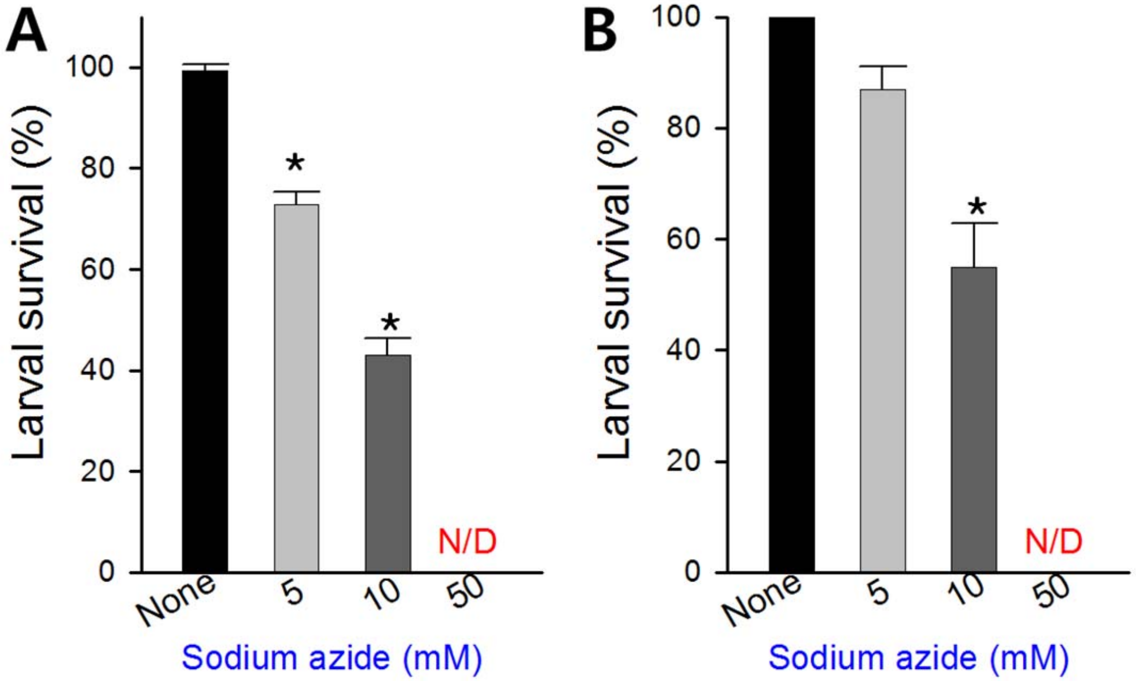

Supplementary Figure S4. Effect of blue and UV LED lights on *B. xylophilus* and *C. elegans* treated with 10 mM sodium azide for 24 h. After 24 h the nematodes were rem oved from the sodium azide solution and placed in sterile distilled water or M9 buffer f or 30 min for recovery. Scale bar: 50 μm.

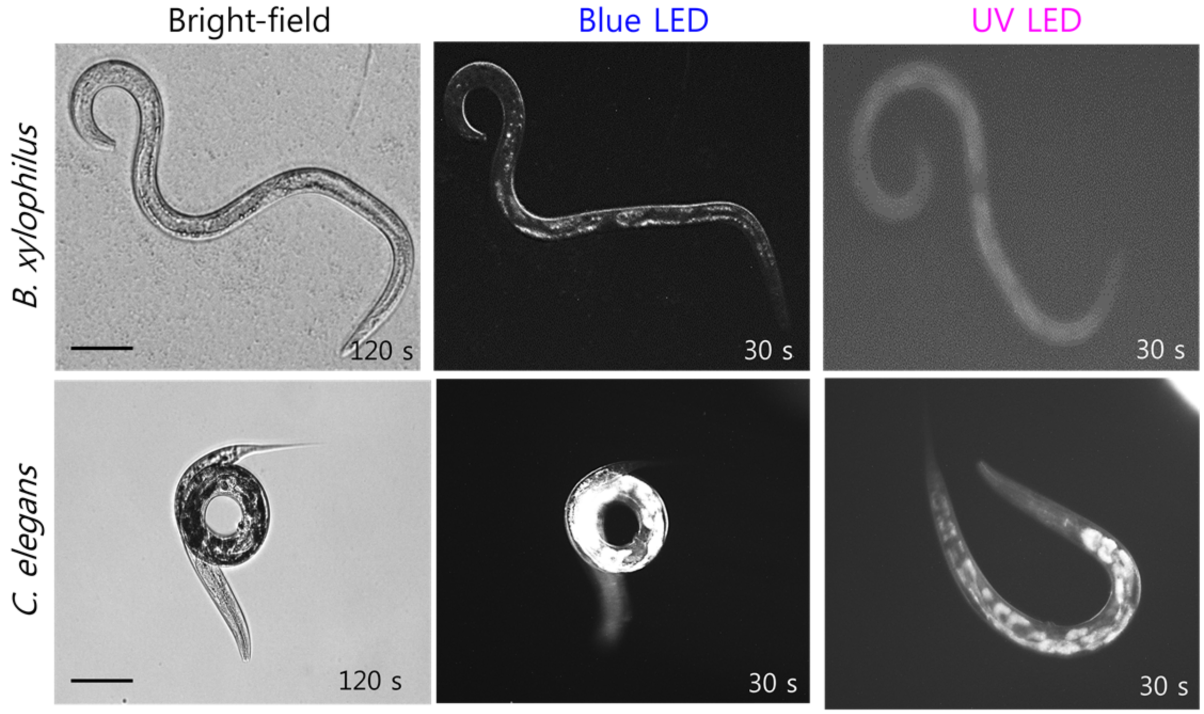

Supplementary Figure S5. The representative image below is showing the effect of stan dard nematicides (25 μg/mL) namely levamisole, ivermectin, and abamectin on synchroniz ed larval stages of *B. xylophilus*. The nematodes treated with levamisole appeared coiled, while the groups treated with abamectin and ivermectin appeared straight. Insets show a single larva with the observed phenotype. Scale bar: 100 μm.

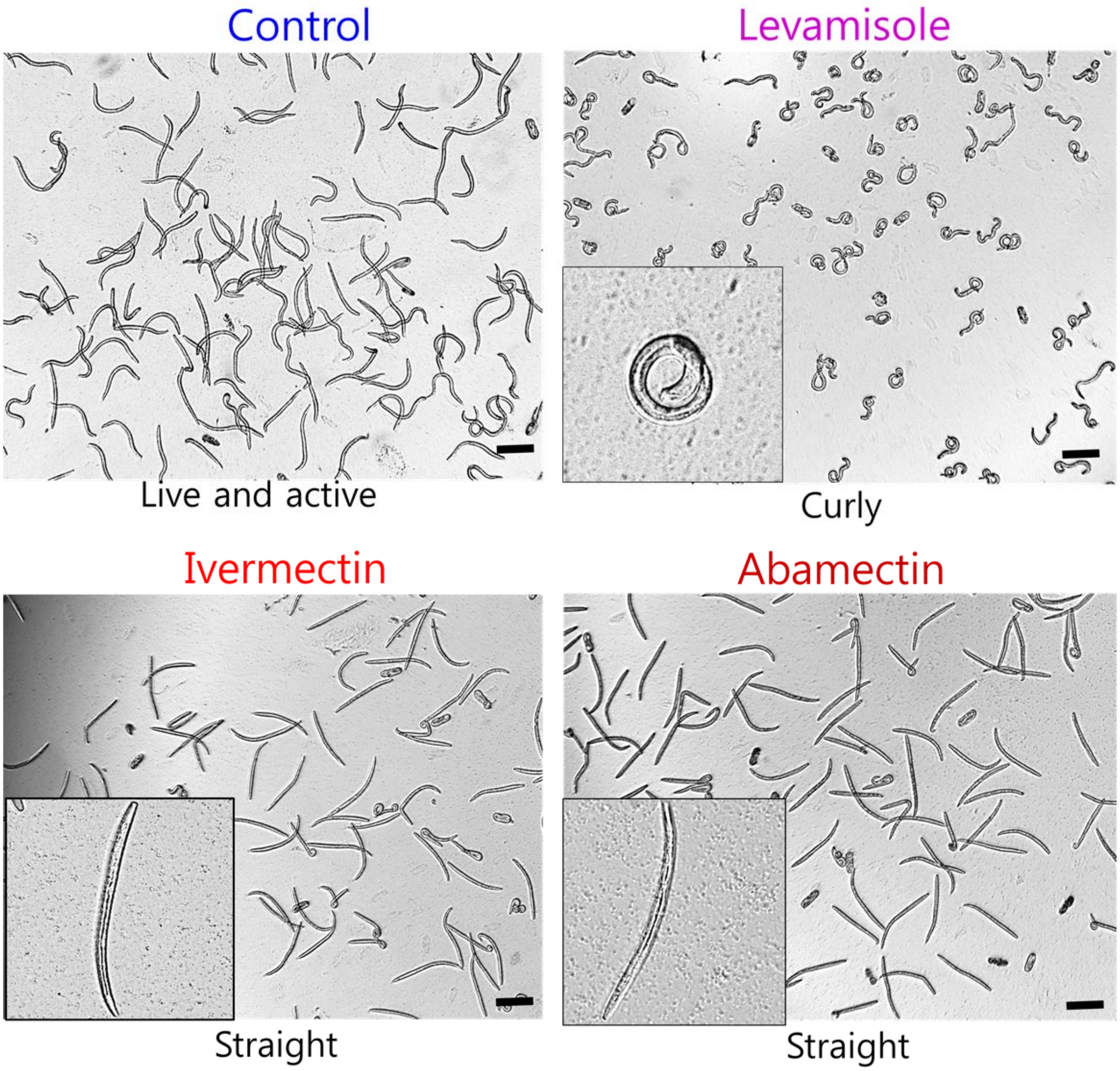

Supplement: Supplementary file 1 — Supplementary information [file 41598_2018_30016_MOESM1_ESM.pdf]
